# Knowledge and Perspectives on the Welfare of Italian Heavy Pigs on Farms

**DOI:** 10.3390/ani11061690

**Published:** 2021-06-06

**Authors:** Marika Vitali, Eleonora Nannoni, Luca Sardi, Giovanna Martelli

**Affiliations:** Department of Veterinary Medical Sciences, University of Bologna, 40064 Bologna, Italy; marika.vitali4@unibo.it (M.V.); luca.sardi@unibo.it (L.S.); giovanna.martelli@unibo.it (G.M.)

**Keywords:** animal welfare, swine, heavy pigs, protected designation of origin, farming, pig health, behavior

## Abstract

**Simple Summary:**

Italian heavy pigs are characterized by much higher body weights and age at slaughter (approximately 160–170 kg, and over 9 months of age) than the majority of pigs reared in Europe. This results in peculiar behavioral and rearing-related needs compared to smaller pigs. However, there is a limited body of research dealing with the welfare of this productive category. The aim of this review is to collect the most significant available information on welfare issues of heavy pigs on farms and to highlight recent findings and areas needing additional research. It is hoped that these findings will, in the future, serve as a basis for the development of specific policies aimed at enhancing the ethical attributes of this renowned production.

**Abstract:**

This review aims to give an overview of the most significant available information on welfare issues of Italian heavy pigs on farms. These animals, whose meat is used to produce typical products, are characterized by much higher body weights and age at slaughter (approximately 160–170 kg, and 9 months of age) than most pigs reared in Europe, resulting in peculiar behavioral and rearing-related needs. To highlight the main findings and the areas in which additional research is needed, the paper was organized by allocating the studies on the basis of the Welfare Quality four principles framework, i.e., good feeding, good housing, good health, and appropriate behavior. The results indicate the possibility of improvement with regard to the management of feeding, watering, and some environmental parameters (light, thermal comfort, enrichments) and a lack of knowledge on the actual space requirements (and a corresponding regulatory gap). Moreover, deficiencies concerning the prevalence of injuries and disease and the relationship between lesions observed post-mortem and rearing conditions needs to be addressed. Lastly, the absence of research concerning the evaluation of the emotional state of animals has been highlighted. It is hoped that these findings will, in the future, serve as a basis for the development of specific policies for these animals in order to increase the ethical value of the entire production chain, in accordance with consumers’ demand and expectation for high animal welfare standards.

## 1. Introduction

Protected Designations of Origin (PDO) and Protected Geographical Indications (PGI) labels represent the excellence of European agricultural food production and are both the result of a unique combination of human and environmental factors that are characteristic of a specific territory. Consumers’ expectations on these products have been increasingly including also implicit ethical attributes, such as environmental sustainability and animal welfare [1,2].

Among the pork-based Italian PDO products, the most well-known are the cured hams, such as the worldwide appreciated Parma ham and San Daniele ham. These types of productions constitute the majority of Italian pork manufacturing. For example, the production of Parma Ham (which is the largest one) is estimated in 2020 with a production value of EUR 720 million, a revenue from consumer sales of EUR 1.500 million, and exports amounting to 29% of the 8.7 million hams produced [3].

According to EU Regulations 1151/2012 [4] and 668/2014 [5] on quality schemes for agricultural products and foodstuffs, in order to be labeled as PDO or PGI these specialties must comply with specific rules throughout the entire production cycle. Taken as a whole, this broad regulatory framework is aimed at obtaining specific high-quality characteristics, which are mainly related to compositional and sensorial attributes of the cured hams.

As an example, Parma ham specifications [6] concern:

- age of pigs (more than 9 months) and body weight at slaughtering (average per batch: 160 ± 10% kg). These requirements imply that males need to be castrated to preserve meat quality;
- genetics: pigs must derive only from specific genotypes included in the production specifications (e.g., Italian Large White, Italian Landrace, Italian Duroc crossbreeds and hybrids deriving from cross-breeding programs with aims consistent with those pursued by the Italian Herd Book for the production of heavy pigs);
- type and quantity of feedstuffs allowed (all the raw materials that can be used in the diets in the different phases of the production cycle and their inclusion levels are explicitly listed. The requirements relating to a minimum age at slaughter and a maximum weight per batch determine the need for moderate weight gains. The animals must therefore receive a rationed feed);
- quality of the raw thighs (the so-called “green hams”) and meat processing techniques (preparation and curing of the thighs, main qualitative parameters of the final product);
- traceability along the whole production cycle (since January 2020, it has been fully computerized).

Concerning animal welfare, the specifications do not include any additional provision other than those already in force by the European legislation on the protection of pigs [5] even though these animals may require additional care due to their heavier weight at slaughter and the longer duration of the rearing period. Moreover, according to a recent audit from the EC [7], few data exist about the welfare conditions of Italian pigs and the presence of implementation strategies. On the contrary, data deriving from a study carried out in the Bologna area (north Italy) indicate that the majority of consumers tend to attribute to PDO products a particularly high content in terms of animal welfare [2]. This observation may indicate that consumers expect PDOs to have multiple qualitative traits, including ethical ones.

This review aims to provide, through an overview on the most significant applicative studies on Italian heavy pigs’ welfare, information and suggestions that may, in the near future, be taken into consideration to increase the ethical value of these renowned productions, in accordance with consumers’ beliefs and expectations.

Additionally, considering that other European and worldwide countries have been increasing pigs’ slaughtering body weight over the past decades to dilute fixed costs [8], the investigation over the welfare level of Italian heavy pigs could be beneficial also under other production contexts.

## 2. Methods

The following review considers studies focusing on the welfare of Italian heavy pigs. A literature search was carried out by consulting the databases ‘Web of Science’, ‘PubMed’, and ‘Scopus’. No date limit was set. The search keywords initially used were “welfare” and “heavy pigs”. The research returned 36 papers on Scopus, 16 on PubMed, and 33 on Web of Science. Duplicate papers were removed. Papers on welfare during transport and pre-slaughter phases were also removed as they did not fit within the scope of this review. Due to the limited number of papers retrieved, when necessary, additional searches were carried out on specific aspects. The reference list from the selected papers was also screened to identify possible additional papers to be included in the present work. As a result of this process, a total of 29 papers about heavy pigs was retained and will be discussed in the present review. The collected studies will be presented in the following paragraphs, divided according to the four welfare principles as described in the Welfare Quality protocol [9] and suggested by the European Food Safety Authority (EFSA) [10,11], namely: good feeding, good housing, good health, and appropriate behavior. For some of the principles (e.g., “good health”), the review includes also studies on the post-mortem assessment of on-farm animal welfare (i.e., assessments carried out at the slaughter plant).

## 3. Good Feeding

### 3.1. Absence of Prolonged Hunger

During the entire growing-fattening phase (approximately 6 months), pigs are traditionally liquid-fed twice a day. The liquid feed is obtained by mixing the commercial feed with water or milk whey, usually in a 3:1 water: feed ratio (up to 1:5 ratio when milk whey is used). Studies on lighter pigs show that when the traditional 3:1 water-to-feed ratio is used, the daily allotment of liquid feed is generally considered sufficient to satisfy both the nutritional and the water requirements [12]. It is also considered sufficient to stimulate a sense of satiety, while higher dilutions (4:1 and above) may reduce dry matter intake and weight gain [13]. According to Hurst et al. [14], liquid feed at 3:1 water-to-feed ratio gave also the best performances in terms of average daily gain (ADG) and feed conversion ratio (FCR), as compared to pellet feeding, in conventional growing-finishing pigs.

The guidelines of the Parma Ham Consortium require pigs to be slaughter at over 9 months of age and average body weight (BW) of 160 kg ± 10% per batch. Therefore, feeding Italian heavy pigs has the purpose to obtain mature animals within a specific body weight range, and this implies restricting feed and energy intake in the diet [15]. This last statement can be particularly relevant with some genetic lines that are characterized by high production efficiency. Restricted feeding can generate an empty stomach, possibly resulting in a drop in gastric pH and an increased risk of oesophago-gastric lesions (OGLs) [16].

Oesophago-gastric lesions are severe and common issues in finishing pigs and they can be responsible for pain, retarded growth, and sudden death in the affected animals [17]. According to Gottardo et al. [18], in heavy pigs, oesophago-gastric ulcers (OGUs) showed a great variation between 120 farms of heavy pigs, ranging from 0% to 36% of prevalence, with 20% of mild-severe ulcerations, and with some farms being able to manage and to avoid them. The study by Di Martino et al. [19] evidenced a reduction of OGUs in groups of pigs receiving continuous straw provision inside a rack during the growing-fattening period, due to the buffering effect of straw on the gastric pH, as reported by other studies [19,20]. Reduced OGUs have been correlated also to a reduction of tail lesions [19,21] and associated with positive effects of anthelmintic treatments and stability of the groups [18], demonstrating their importance in the welfare of pigs.

### 3.2. Absence of Prolonged Thirst

Not many studies have addressed the water requirements of Italian heavy pigs. However, the availability of drinkers has been recently included in the Italian protocol for welfare assessment [22] released by the Italian Ministry of Health (ClassyFarm, [23]). As stated above, in Italian heavy pig production, liquid feeding is the most common technique in the growing-finishing stage. In northern and northeastern regions of Italy, the historical availability of whey as a by-product of cheese production (e.g., Parmigiano Reggiano and Grana Padano) has encouraged liquid feeding as a widespread practice because of its relatively low costs. Liquid feeding was also considered a method of reducing the volume of farm effluents, and since the daily allotment of water provided in this way was considered sufficient, additional drinkers were not always provided. Some farmers adopted the practice, especially during the hot season, to distribute additional water in the feeding trough once a day, between the two meals.

According to the study by Nannoni et al. [24], if environmental temperatures are moderate (during the winter season) and no illness occurs, liquid-fed pigs (3:1 water: feed ratio) offered supplementary water from the drinkers do not show growth or behavioral parameters changes compared to animals provided with non-working drinkers. Animals, however, accessed the non-working drinkers throughout the trial, indicating that the motivation to obtain additional water may persist even when the theoretical water requirements have been fulfilled by the daily liquid fed ration. According to Vermeer et al. [25], the demand for additional water by liquid-fed pigs did not differ from dry-fed pigs, suggesting a similar motivation to drink water. This would indicate that, in theory, and on average, the need for water could be met via the intake of liquid feed. However, when time-related variations occur for a given pig, such as physiological variations due to individual differences or environmental conditions, the water requirements can significantly increase [25]. For example, some studies measured that under heat stress, the need for water can double [26]. In this latter case, the frequent use of drinkers has the purpose to foster heat dispersion from the skin and help thermoregulation. It is reasonable to say that with very high temperatures (such as summer temperatures in south Europe) and insufficient environmental control, the presence of additional drinking water can help the animals to cope with the thermal stress, also if the more efficient solution should be adopted (e.g., cooling systems) to avoid wasting water. Overall, studies [24,25] agree that despite the theoretical needs could be met by the daily water allotment of liquid feed, the animals would still benefit from the provision of fresh drinking water. This aspect has been highlighted also by a recent (March 2021) Italian Ministerial Note, who stated that no derogation is permitted from the presence of drinkers, regardless of the feeding method [27].

## 4. Good Housing

### 4.1. Comfort around Resting and Ease of Movement, also in Relation with Thermal Comfort

Floor space availability certainly represents the design criteria that has the greatest impact on freedom of movement and comfort around resting of intensively-reared pigs, consequently influencing their level of welfare and productivity [28,29]. Besides the need for a “personal space”, in order to avoid the constant physical contact with other animals, pigs also require space to rest (need of a static nature) and to move (need of a dynamic nature which also includes social activities). Granting an adequate space allowance is extremely important for heavy pigs since, in the 100–160 kg BW range, they spend most of their time (69.7% of the daily hours and 97.7% of the night hours) lying in a resting position (either sternal or lateral recumbency) [30].

Pigs tend to use separate areas for the different activities of resting, feeding, defecating, and urinating. If this division of the pen in different functional areas cannot be obtained due to the lack of space or to design errors, there is a risk that overlaps will occur between the areas (for example, between the rest area and the defecation area) resulting in a decline in the level of animal welfare. In this case, “dirtiness” (soiled pigs) could be used as a parameter that would reflect the negative effect of lack of space on animal welfare [31].

Environmental conditions can also influence the use of space and its related need: at high temperatures, pigs must be able to lie down in lateral recumbency, avoiding physical contact with pen-mates, thus leading to a need for additional space to cope with the housing system. Another important parameter to consider is represented by the size of the pen: small groups of animals (4–10 pigs) require, in proportion, more space than larger groups [32]. European legislation [33], while providing for a progressive increase in space as the live weight of pigs increases, sets to 1 m^2^/head the minimum floor space allowance for pigs over 110 kg body weight (BW) without further requirements for heavier animals, with the only exception for organically reared pigs (minimum indoor area of 1.5 m^2^/head for pigs weighing more than 110 kg [34]), that, however, account only for a very limited number on the total volume (less than 1%) [35]. These minimum requirements are calculated after the equation A (minimum space allowance) = 0.030 × BW^0.67^, where the coefficient of the equation (k) is set to 0.030 [29]. However, according to EFSA recommendations [32], higher coefficients should be used (k = 0.036 for pigs up to 110 kg and k = 0.047 above 110 kg) in order to allow all pigs to lay down separately in lateral recumbency and at the same time. Despite this broad set of considerations, to our best knowledge, only very few studies have specifically dealt with the space requirements of the heavy Italian pigs, which, besides their very high live weight at slaughter, are reared in geographical areas where the maintenance of thermal comfort may not always be effectively guaranteed.

In a first trial, Rossi et al. [36] compared the effect of two different floor space allowances: a low space allowance (LSA; k = 0.033; 1.0 m^2^ per animal; 126 pigs) and a high space allowance (HSA; k = 0.047; 1.4 m^2^ per animal; 90 pigs) in the body weight range of about 90 to 156 kg. Pigs on all floor types tended to gain more weight with HSA than with LSA during the last phase of the production cycle. Feed conversion ratio tended to be lower for HSA than LSA animals. Backfat thickness was higher for HAS. Although these results are of great scientific interest, they fail to fully dispel doubts about what is the optimal floor space availability for heavy pigs. The larger available surface (LSA) was, in fact, obtained by decreasing the number of animals per pen and not by increasing the space (i.e., the number of pigs per pen was not kept constant), therefore the two effects could not be evaluated separately. Accordingly, the authors define the treatment as “group size-space allowance” and do not refer to the effect of the space as such. Interestingly, a further study from the same research group [37] supports spatial provisions of Directive 120/2008 [33] for space needs in the BW range of 115–150 kg, as assessed by direct measure of animals and digital image analysis. However, these results (direct measures and digital imaging) are below the theoretical spatial requirements obtainable by using the coefficient k = 0.047 and are based on a static approach that does not take into account the spatial needs for functional areas separation and social behavior. These aspects could be the object of future studies.

More recently, Nannoni et al. [38] compared the effect of different floor space availabilities in a BW range from 24 to 160 kg. Pigs were kept in small groups of 5 animals at 1.0 or 1.3 m^2^/head (corresponding to a k = 0.047 for pigs of 135 kg BW). Under the experimental conditions, pigs raised at 1.3 m^2^/head were calmer and spent more time laying (particularly in lateral recumbency), reduced the aimless exploration of floor and increased the overall expression of other, mainly active, behaviors. Pigs reared at 1.3 m^2^/head showed also higher final body weight and average daily weight gain, and a more favorable feed conversion ratio. No significant difference was observed between groups for carcass traits and the main meat quality attributes. Despite these interesting findings, also in this case, it is not possible to conclude on space requirements of heavy pigs. The low number of animals per box (*n* = 5), is, in fact, a highly limiting factor for the transposition of the results to the common rearing practice. In fact, the group size can influence the welfare outcome and should be taken into account when considering the effects of space allowance. Smaller group sizes allow an easier establishment of the hierarchy and therefore they can reduce fighting after mixing [39]. Further, a very large group size has been found to reduce fighting, since it stimulates the creation of sub-groups of pigs [40]. Additionally, management practices such as the frequency of group mixing need to be considered. Stable groups throughout rearing have been related to a lower risk of injuries (e.g., skin lesion, lameness) [39]. Overall, the examination of the few available papers on the spatial needs of Italian heavy pigs does not allow to reach firm conclusions.

Another aspect related to good housing and deserving consideration is flooring, since it can affect animal comfort, health, and presence of lesions. Italian heavy pigs are generally raised indoors on either fully slatted or partially slatted floors. In consideration of their high weight at slaughter, the Italian Ministry of Health allows the adoption of fully slatted floors with a maximum width of the openings of 20 mm (2 mm more compared to the EU Directive on pig protection [33]) and a minimum width of the slats of 80 mm [41]. In general, older pigs have been reported to be at higher risk to develop bursitis because their greater body weight exerts additional pressure on the limbs and they spend a greater proportion of time lying [42,43]. Bursitis in heavy pigs was assessed, to the best of the authors’ knowledge, only by Bottacini et al. [42] (observation were made after slaughter by observing only the front limbs, due to technical constraints), finding a maximum batch prevalence of 10%. This prevalence already represents a welfare concern. Moreover, the authors reported that examining only the forelimbs may have underestimated the actual prevalence since bursitis is reported to occur more frequently on the hind legs.

Slatted floors have been also associated with an increased prevalence of injuries and lameness in pigs, because they provide the animals with many particular challenges (uneven walking surface, reduced weightbearing surface, lack of bedding, and sharp slat edges) [43]. To our knowledge, no study specifically measured lameness prevalence in Italian heavy pigs on farms, but these parameters should be monitored to assess the effect of flooring characteristics on pigs.

### 4.2. Light Requirements

Mandatory levels of environmental illumination for pigs are set by the European legislation on pig protection [33] to a minimum of 40 lux for at least 8 h/d. This provision reflects the existence of a need of pigs in terms of light intensity and duration, which must be fulfilled to allow their explorative and social activities [44]. Literature dealing with pig requirements in terms of environmental illuminance is not abundant and, in some cases, contradictory due to a certain degree of confusion between daylight duration, light intensity, and light source (natural/artificial). To the best of the authors’ knowledge, no official data exist about light provision and management in Italian heavy pig farms. In the past, keeping pigs in semi-darkness was considered effective to reduce aggressive behaviors by some Italian farmers. A series of studies were conducted at the Department of Veterinary Medical Sciences of the University of Bologna (Italy) to identify the needs of Italian heavy pigs in terms of duration and intensity (illumination was provided by neon tubes) of the artificial photoperiod throughout the entire growing-fattening phase. A first study [45] compared the effects of two lighting durations, i.e., 14 and 8 h/day, at a light intensity of 70 lux. A second experiment [46] was aimed at investigating the effects of two light intensities, i.e., 40 and 80 lux (artificial light phase of 12 h). A third trial [47] compared the effects of photoperiods of different duration at a high light intensity (14 vs. 8 h of light phase with a 70-lux intensity) and the effects of two light intensities (40 and 80 lux; artificial light phase of 12 h). Lastly, a fourth research [48] investigated the effects of a long illumination period of 16 h/d at low light intensity (40 lux). The end points were, depending on the study, growth parameters, animal behavior, carcass, meat, and hams (raw and cured) quality.

The outcomes (Table 1) indicate that an artificial photoperiod longer than the minimum set by legislation (i.e., more than 8 h of light-phase per day) resulted in more favorable daily weight gain and feed efficiency [45,48]. On the other hand, growth parameters were not affected by the light intensities tested [46]. Neither the duration of the lighting period, nor the light intensity caused any negative changes in carcasses, meat, and hams (raw and cured) quality [46,47,48]. A light intensity above the minimum set by legislation resulted in a higher percentage of observations indicating resting behavior (i.e., higher calmness degree) [45,48]. A higher level of light was associated with a lower number of aggressive interactions between animals [46].

Taken as a whole, these results suggest that, given an appropriate dark period for animal rest of a least 8 h of darkness, increased duration of the photoperiod and/or of light intensity above the minimum mandatory levels can favorably affect growth parameters and welfare level of heavy pigs. Welfare improvement may probably be due to lower levels of discomfort and agitation resulting from a better ability to recognize the surrounding environment and to cope with it, leading to the increased time spent resting.

Without detracting from what has just been reported regarding the light requirements of heavy pigs (in terms of both duration and intensity), a recent study by Marinelli et al. [49] showed that under specific situations, such as when new groups of animals need to be formed, a 48-h period of darkness can reduce the severity of skin lesions resulting from fighting.

## 5. Good Health

### 5.1. Absence of Injuries

Most studies on heavy pigs were conducted under experimental conditions and/or considered only one farm, therefore there is a lack of epidemiological knowledge on the prevalence and type of injuries across farms.

To our knowledge, only the study by Scollo et al. [50] recorded on-farm the prevalence of lesions from tail biting in 67 farms of heavy pigs. The sample included 39 weaning sites and 28 fattening sites. All pigs were tail-docked. The results showed that, on average, the prevalence of tail lesions was higher in the fattening sites (0.26%) compared to weaning sites (0.09%). The overall prevalence was very low, but the assessment has been conducted from outside the pen. The study identified some risk factors for tail lesions, such as tail length, adverse air quality (as perceived by the observer), and timeliness in feed supply, in weaning sites. In the fattening sites, only stocking density was statistically associated with tail lesions.

The scarcity of studies about on-farm lesions highlights the need for further research on this topic. Assessing skin and tail lesions on the carcasses would be more practical and feasible. The study by Vitali et al. [51], conducted on 79 batches of tail-docked heavy pigs, observed tail lesions on 34.0% of the carcasses, of which only 4.4% were severe lesions (open wounds and/or loss of tissue) [51]. The same authors reported high differences between this result and previous studies on tail lesions in tail-docked Italian heavy pigs [42,52], mainly imputable to different scoring systems and place of observation [53].

Bottacini et al. [42] proposed to use chronic ear lesions, tail lesions (defined as the presence of notches, necrosis, bites, or scars), and bursitis, all assessed at slaughter, as retrospective indicators of the on-farm welfare conditions of heavy pigs. The results of the study showed the highest prevalence of ear lesion referred to pigs reared during the spring, whereas tail lesions did not show any seasonal fluctuation. Unfortunately, bursitis and ear lesions showed also an increased prevalence in batches undergoing overnight lairage, enlightening the need to distinguish between on-farm and pre-slaughter lesions to avoid misinterpretations.

### 5.2. Absence of Disease

The majority of studies on disease prevalence in Italian heavy pigs has been conducted using post-mortem assessments. This can be explained, as the monitoring of health at slaughter involves assessments that cannot be performed at the farm, for example, lesions of the pluck (lung, pleura, and liver). The prevalence of gross anatomic lesions can then be linked retrospectively to on-farm conditions, or data can be used for an epidemiologic investigation on health and disease.

The prevalence of pluck lesions (i.e., enzootic pneumonia-like lesions, pleuritis, pericarditis, and white spots on the liver) has been recorded in some studies on heavy pigs [51,54,55,56,57], with the purpose to assess lesion prevalence and to benchmark and monitor the occurrence of diseases on farms. In these studies, data were used for epidemiological purpose and health surveillance, or as a monitoring tool with the purpose to provide farmers with feedback on herd health conditions. For example, a recent study monitored pluck lesions and found a 30.2% prevalence of enzootic pneumonia-like lesions, showing a reduction trend compared to previous data retrieved from the literature in the same category of pigs [51,55]. The prevalence reduction was likely tied to the diffusion of vaccination against *M. hyopneumoniae*. On the contrary, the prevalence of dorsocaudal chronic pleuritis, suggestive of infection by *A. pneumoniae*, did not show any reduction [54]. This type of feedback should be considered in the management plan of the farms, to improve the efficiency of the production cycle. It is important to consider that, except for Merialdi et al. [54], no association studies have been carried out between pluck lesion assessed after slaughter and herd factors. In this study, a reduction in enzootic pneumonia lesions has been associated with PRRS (Porcine Reproductive and Respiratory Syndrome) vaccination, while chronic pleuritis did not show to be influenced by any of the tested farm conditions (i.e., farm dimension, ventilation system, floor type, farrow-to-finish system; all-in/all-out procedures). Further studies will be necessary to provide a more complete report for the farmers, helping in the accurate identification of risk factor affecting pig health and welfare. Pluck lesions are mainly indicative of infections in the post-weaning phase, although it has been hypothesized that, due to the long production cycle and to the challenging conditions in the intensive production of heavy pig (e.g., low space allowance, unfavorable microclimate, high gases and dust concentration) in the late rearing phase, could interfere with the healing process or facilitate co-infections (e.g., with Porcine Respiratory and Reproductive Virus, Influenza virus) [55].

It is important to report that the recently introduced Italian welfare assessment protocol, ClassyFarm (see Section 5.3) will permit to collect animal-based measures (ABMs) also at the slaughterhouse, thus allowing the monitoring of health and welfare indicators along the pig production chain [58].

### 5.3. Absence of Pain Induced by Management Procedures

According to Directive 120/2008 EC [33], mutilations should be avoided because they cause pain and suffering to the pigs. Some exceptions are allowed, such as castration for animals intended for typical products, or in case alternative procedures are not available. In heavy pig production, two types of mutilation are still practiced: castration and tail docking.

Castration of male piglets is mainly aimed at reducing the occurrence of boar taint in meat. At present, surgical castration is the most frequently applied method and it generally takes place within the first week of life, often without the aid of painkillers and/or anesthesia [59], causing acute or chronic pain and distress in piglets [60]. In 2010, the European Declaration on alternatives to surgical castration of pigs, recommending the abandonment of surgical castration by 2018, was signed [61]. In consideration of the high age at slaughter and of the need for excellent quality thighs, castration is an unavoidable practice for the production of Italian heavy pigs intended for PDO hams. In this framework, immunocastration, which consists in a vaccination against GnRH (Gonadotropin Releasing Hormone), may represent a viable alternative to surgery. From a practical standpoint, it is worth noting that, due to the higher age and longer lifespan, Italian heavy pigs need an additional dose of vaccine compared to lighter animals (3 vs. 2 doses). This results in higher costs and greater operational difficulties due to the administration of the product by injection to animals of high weight, which are more difficult to handle. The effects of immunocastration on the quality of the meat of heavy pigs (Landrace*Large White crossbred) have been investigated by Pinna et al. [62], who concluded that three-dose immunocastration may be a reliable alternative to surgical castration. However, the authors stressed that the topic deserves more investigation, especially into other genotypes and commercial cuts.

As for consumers’ acceptance of meat from immunocastrated pigs, a recent survey carried out on a representative sample of the Italian population [63] indicated that immunocastration is perceived in a predominantly positive manner (54.5%), with a relatively low level of risk perception (34.2%), and a good willingness to pay more for meat deriving from immunocastrated pigs (+18.7%). These outcomes are confirmed by a further study involving respondents from several European countries, including Italy [59], that showed, even though with marked differences among nations, that castration with anesthesia had the highest acceptance (85%), followed by immunocastration (71%) and production of entire males (49%) while the practice of surgical castration was the least accepted (32%), mainly due to animal welfare concerns. These results may indicate that, once all scientific doubts have been resolved, immunocastration could become a common practice also in the production of Italian heavy pigs, with clear benefits in terms of animal welfare.

The other mutilation procedure that is common worldwide and also in heavy pig production is tail docking, which is carried out with the purpose to prevent tail lesions derived by tail biting behavior. However, According to the Directive 120/2008 EC [33], this practice should not be carried out on a routine basis unless other preventive measures proved to be ineffective.

An audit carried out in Italy by the European Commission on the Member States’ activities to prevent tail-biting and avoid tail-docking [7] reported that until 2017, tail docking was routinely performed in almost 100% of pig farms, and that no measures for benchmarking tail lesions or risk assessment protocols were used. Similar situations have been found in other 20 Member States according to Nalon et al. [64]. Following this assessment, in 2019, the Ministry of Health launched ClassyFarm [22], an integrated risk assessment protocol. Its first application was in pigs, with the purpose of monitoring health and welfare conditions in farms, suggesting also corrective measures to increase welfare and to avoid the need for tail docking. Moreover, the Ministry recommended that farmers should rear pilot groups of pigs with intact tails, defining progressive goals which have the potential to drive the production towards the abandonment of routine tail docking and the accomplishment of the European standards [65]. Even if farmers are concerned about the abandonment of tail docking [66], only a few studies have been performed on rearing heavy pigs with intact tails in commercial farms. Di Martino et al. [67] investigated the effect of tail docking in heavy pigs undergoing stressful conditions, highlighting that fattening undocked pigs showed a higher prevalence of moderate tail lesions as compared to tail-docked pigs, and that severe tail lesions and mortality rates did not differ between the two groups, as well as blood physiological indicators.

Based on these encouraging results, it is certainly necessary to promote the study of strategies aimed at reducing aggressive behaviors among undocked pigs, some of which are discussed in the following Section 6.1 (“Expression of behaviors: environmental enrichment”).

## 6. Appropriate Behavior

### 6.1. Expression of Behaviors: Environmental Enrichment

Heavy pigs, similarly to the majority of pigs raised for food production, are usually kept indoors, in a barren environment that does not meet their need to express species-specific behaviors (such as rooting) and does not fulfil their intrinsic motivations. Because of that, the permanent provision of manipulable materials is explicitly listed among the requirements set out in the EU Directive on the protection of pigs [33]. In addition, the Commission Recommendation 2016/336 [68] states that enrichments should be edible, chewable, investigable, manipulable, of sustainable interest, accessible, given in sufficient quantity, and clean. Materials possessing all these characteristics are defined as ‘optimal materials’ and can be used alone, whereas ‘suboptimal materials’ (i.e., materials possessing most of the characteristics listed, but not all) should be used in combination with other materials. On the basis of the above-mentioned characteristics, straw litter can be considered as a very effective (optimal) enrichment, able to increase satiety and avoid tail biting outbreaks [69,70]. However, since most heavy pigs are kept on totally/partially slatted floors, straw use can interfere with slurry-removal systems, thus making it necessary to chop it or make it available in racks. Because of that, according to a questionnaire sent in 2011–2013 to 162 farms in Italy [71], many farmers resulted to prefer hanging chains as an enrichment tool, and many of them had doubts about the actual effectiveness of environmental enrichments themselves.

With regards to the effectiveness of enrichment tools on heavy pigs, the results of some studies concerning different types of environmental enrichments made available to heavy pigs are summarized in Table 2. According to Scollo et al. [72], long straw available at all times in a metal rack attached to the wall increased the motivation for exploring, reduced serum haptoglobin and tail lesions in undocked heavy pigs. Therefore, straw seems to be an important tool in both increasing explorative behavior and preventing biting and lesions, particularly in the early stage of the growing-fattening phase (weeks 3 to 18 over a 30-week fattening period starting at approximately 80 d of age). Moreover, the consumption of small amounts of straw (70 g/day/pig) represents a protective factor against the onset of oesophago-gastric ulcers in undocked heavy pigs [73].

Concerning undocked heavy pigs reared on fully slatted floor, Nannoni et al. [74] compared the effect of edible blocks (100 × 30 × 10 cm^3^; optimal enrichment material) and wood logs (suboptimal enrichment material) placed into metallic racks. In both cases, hanging chains served as control. No differences were observed in growth parameters, hair cortisol concentration, neutrophil/lymphocyte ratio, bristle cortisol, and skin lesions at the end of the trial (although pigs receiving wood logs showed a higher prevalence of mild tail lesions at some time points during the trial). Edible blocks were more attractive than hanging chains which, in turn, were more attractive than wood logs. Small, although significant, differences were noted for the quality of the carcasses which, however, did not result to have a commercial impact. Both edible blocks and wood logs did not modify the prevalence of gastric lesions compared to control pens provided with metal chains [21]. The basic similarity of the results from animals receiving different enrichments in this study could indicate that under appropriate rearing conditions (small and stable groups, higher space allowance, good human–animal relationship), a good level of animal welfare can be achieved. With respect in particular to tail biting, since its prevention is a combination of enrichment provision and control of several other risk factors, it should be highlighted that there is a lack of papers addressing how to evaluate these risk factors (and their interactions with enrichment provision) on-farm, especially for animals which are raised for prolonged periods. Therefore, these observations should be confirmed by further research on the topic.

### 6.2. Positive Emotional States and Good Human–Animal Relationship

Promoting positive welfare is attracting considerable research interest, because it considers the emotions of the animals, or rather their behavioral, neurophysiological, and cognitive components [75,76].

It is worth noting that negative emotions such as fear, distress, and anxiety could have a large impact on the physiology, behavior, and health of animals, making the study of emotions a key step in animal welfare research [77]. In intensive livestock production, many welfare problems can originate from inadequate stockmanship [78]. Accordingly, the human–animal relationship has been considered a pivotal issue in pig production [79].

Because of that, the evaluations of animals’ emotional state and human–animal relationship have been included in some welfare assessment protocols, including Welfare Quality [9].

To the best of the authors’ knowledge, no studies have investigated the emotional state and the human–animal relationship in Italian heavy pigs [23]. Therefore, this information is still largely unknown in this type of production. Assessing affective states using valid tools such as qualitative behavior assessment (QBA) or fear tests might provide meaningful information on the pig herd, and be helpful in the identification of welfare issues, also considered the welfare challenges described in this review that these animals may face and the large impact of repeated and/or chronic stressors on their emotional state.

## 7. Conclusions

There is only a limited number of studies available on the welfare level and the welfare assessment of Italian heavy pigs, and several aspects have not been investigated yet. In particular, the lack of knowledge on space requirements, injuries, and positive welfare (included human–animal relationship) are crucial aspects that should be explored in order to define a baseline to set up measures for the improvement of the production system. It is hoped that this review will help to promote specific policies aimed at enhancing the ethical attributes of this renowned production, thus improving animal welfare, meeting consumers’ expectations, and increasing the value of its marketing chain.

## Figures and Tables

**Table 1:** Summary of the main findings from studies on illumination requirements of heavy pigs.

	ADG	FCR	Meat Quality	Behavior	Aggressive Interactions	Ref.
**LIGHT DURATION (hours per day)**						
**14 vs.8 h (at 70 lux)**	↑	↓	n.s.	↑ lateral recumbency	n.a.	[46,47]
**16 vs. 8 h (at 40 lux)**	↑	↓	n.s.	↑ total recumbency	n.a.	[48]
**LIGHT INTENSITY (lux)**						
**40 vs. 80 lux**	n.s.	n.s.	n.s.	↑ sternal recumbency ↓ drinking	↓	[46,47]

ADG: average daily weight gain; FCR: feed conversion rate; ↑: increased; ↓: reduced; n.s.: not significant; n.a.: not assessed.

**Table 2:** Summary of the main findings from studies on environmental enrichments for heavy pigs.

	Growth Parameters	Blood Parameters	Carcass/Meat Quality	Behavior	Ear Biting	Tail Biting	Gastric Lesions	Ref.
**Straw**	n.s.	↓ haptoglobin	n.a.	↑explorative behavior ↓ lying	↓ risk	↓ risk	↓ risk	[19,72]
**Edible blocks**	n.s.	n.s.	↓ loin thickness	↑ interactions with the enrichment	n.a.	n.s.	n.s.	[21,74]
**Wood logs**	n.s.	n.s.	↑ lean meat% ↓ backfat thickness	↓ interactions with the enrichment	n.a.	↑ moderate lesions but no differences at slaughtering	n.s.	[21,74]

↑: increased; ↓: reduced; n.s.: not significant; n.a.: not assessed.

## Data Availability

Data sharing not applicable.
